# Advanced QbD-Based Process Optimization of Clopidogrel Tablets with Insights into Industrial Manufacturing Design

**DOI:** 10.3390/pharmaceutics17050659

**Published:** 2025-05-17

**Authors:** Young Woo Bak, Mi Ran Woo, Hyuk Jun Cho, Taek Kwan Kwon, Ho Teak Im, Jung Hyun Cho, Han-Gon Choi

**Affiliations:** 1 College of Pharmacy, Hanyang University, 55 Hanyangdaehak-ro, Sangnok-gu, Ansan-si 15588, Gyeonggi-do, Republic of Korea; 2 Pharmaceutical Research Centre, Hanmi Pharm. Co., Ltd., 893-5 Hajeo-ri, Paltan-myeon, Hwaseong-si 18536, Gyeonggi-do, Republic of Korea; 3 College of Pharmacy, Keimyung University, 1095 Dalgubeol-daero, Dalseo-gu, Daegu 42601, Gyeongsang-do, Republic of Korea; 4 Department of Pharmaceutical Engineering, Dankook University, 119 Dandae-ro, Dongnam-gu, Cheonan-si 31116, Chungcheongnam-do, Republic of Korea

**Keywords:** clopidogrel, quality by design, critical quality attribute, intermediate critical quality attribute, critical process parameter, control strategy

## Abstract

**Background/Objectives**: Traditional Quality by Testing (QbT) strategies rely heavily on end-product testing and offer limited insight into how critical process parameters (CPPs) influence product quality. This increases the risk of variability and inconsistent outcomes. To overcome these limitations, this study aimed to implement a Quality by Design (QbD) approach to optimize the manufacturing process of clopidogrel tablets. **Methods**: A science- and risk-based QbD framework was applied to identify and prioritize key CPPs, intermediate critical quality attributes (iCQAs), and final product CQAs across key unit operations—pre-blending, dry granulation, post-blending, lubrication, and compression. Risk assessment tools and statistical design of experiments (DoE) were used to define proven acceptable ranges (PARs). **Results**: The study revealed strong correlations between CPPs and CQAs, allowing the definition of PARs and development of a robust control strategy. This led to improved manufacturing consistency, reduced variability, and enhanced process understanding. **Conclusions**: The QbD approach minimized reliance on end-product testing while ensuring high-quality outcomes. This study offers a novel QbD implementation tailored to the manufacturing challenges of clopidogrel tablets, providing a validated approach that integrates dry granulation CPPs with process-specific CQAs. These results demonstrate the value of QbD in achieving robust pharmaceutical manufacturing and meeting regulatory expectations.

## 1. Introduction

Ensuring consistent product quality has traditionally relied on end-product testing, commonly referred to as Quality by Testing (QbT). While this approach can detect non-conforming batches, it often fails to uncover the root causes of variability due to its limited focus on the underlying manufacturing process [1]. As a result, QbT-based systems can lead to inefficient troubleshooting, higher production costs, and significant financial losses [1,2]. To address these limitations, Quality by Design (QbD) was introduced as a proactive, science- and risk-based approach to pharmaceutical development [3]. QbD emphasizes building quality into the product from the earliest stages of development rather than testing it afterward [4]. The approach is centered around gaining process understanding, identifying sources of variability, and designing robust processes to ensure consistent product performance [5]. The QbD framework begins by defining a Quality Target Product Profile (QTPP), which outlines the intended clinical performance and key product attributes. From the QTPP, Critical Quality Attributes (CQAs) are identified—these are the physical, chemical, biological, or microbiological properties that must be controlled to ensure product quality [6,7]. Based on the CQAs, critical process parameters (CPPs) are identified as key variables that must be optimized to maintain quality throughout the manufacturing process [8,9]. QbD typically incorporates tools such as Design of Experiments (DoE), risk assessment, and multivariate analysis to define a design space and establish Proven Acceptable Ranges (PARs) [10,11]. In addition to enhancing process robustness, QbD supports lifecycle management and regulatory flexibility. It has been endorsed by major regulatory authorities such as the FDA, EMA, and WHO, and forms a central part of ICH guidelines Q8 to Q11 [12,13,14]. As regulatory expectations evolve toward greater process transparency and risk-based decision-making, QbD has emerged as a gold standard for product development and continuous improvement [15].

Clopidogrel, a thienopyridine-class antiplatelet agent, is widely prescribed for the prevention of thrombotic cardiovascular events. However, it presents several formulation and manufacturing challenges, including low aqueous solubility (BCS Class II), sensitivity to polymorphic transitions, and variable dissolution depending on process conditions [16,17,18]. These factors can contribute to poor bioavailability and batch-to-batch variability, making it an ideal candidate for QbD application to improve process control and ensure product consistency.

In this study, we applied a full QbD approach to optimize the manufacturing process of clopidogrel tablets via dry granulation. Each unit operation—from pre-blending to compression—was evaluated using DoE to identify CPPs and their relationships to iCQAs and final CQAs such as blend uniformity, hardness, friability, and dissolution rate [19]. The dry granulation method was selected to minimize the risk of hydrolytic degradation and improve flow properties without using solvents [20]. Key parameters such as roller pressure, gap, and speed in the compaction stage, as well as turret and feeder speed during compression, were studied to determine their influence on tablet performance. Acceptable parameter ranges were identified to define a robust control strategy across the manufacturing process [21,22,23].

Beyond this case study, QbD has been widely applied to various formulation types. Recent studies have demonstrated its utility in solid dispersions [11,24], liposomal formulations [25], biologics [26,27], and nanoformulations [28,29,30]. These representative applications of QbD across different formulation platforms are summarized in Table 1 (Table 1). These examples underscore the versatility of QbD in modern pharmaceutical development, where complex products demand sophisticated control strategies.

However, to our knowledge, no previous study has systematically applied QbD to optimize dry granulation-specific CPPs of clopidogrel tablets while also correlating iCQAs with final CQAs across all unit operations. This integration enables an industrially feasible and regulatory-compliant control strategy, distinguishing our work from existing QbD case studies.

Moreover, the integration of QbD with Process Analytical Technology (PAT) has enabled real-time process monitoring and control, allowing manufacturers to detect deviations and maintain quality during production [31,32]. Continuous manufacturing platforms are also being developed under QbD principles to improve scalability and reduce time-to-market [33,34]. Additionally, recent advances incorporate artificial intelligence (AI) and machine learning (ML) to build predictive models based on historical manufacturing data, further enhancing process understanding and automation [35,36].

In conclusion, this study presents a comprehensive application of QbD to the development of clopidogrel tablets. By linking process parameters to product quality attributes and conducting in vivo bioequivalence testing, we demonstrate how QbD can bridge the gap between manufacturing design and therapeutic performance. This work contributes to both scientific understanding and industrial practice by offering a structured, evidence-based pathway for robust pharmaceutical development.

## 2. Materials and Methods

### 2.1. Materials

Clopidogrel napadisilate monohydrate spray-dried dispersion was supplied by Hanmi Pharm. Co. (Hwaseong-si, Republic of Korea). Fumaric acid (EMPROVE ESSENTIAL) was purchased from Merck (Kenilworth, NJ, USA). D-mannitol (Pearitol^®^ 200 SD) was obtained from Roquette (Lestrem, France). Colloidal silica (HDK^®^ N20 Pharm Co.) was acquired from Wacker (Nunchritz, Germany). Crospovidone (Kollidon^®^ CL) and copovidone (Kollidon^®^ VA 64) were purchased from BASF (Ludwigshafen, Germany). The sucrose esters of fatty acids (DK ESTER F-20W) were supplied by DKS Co., Ltd. (Shiga, Japan). All other chemicals and solvents were of reagent grade and used without additional purification.

### 2.2. Preparation of Clopidogrel Tablets

The detailed composition of the tablet formulation is presented in Table 2. Clopidogrel napadisilate spray-dried dispersion was blended with fumaric acid (EMPROVE^®^ ESSENTIAL), crospovidone (Kollidon^®^ CL), copovidone (Kollidon^®^ VA 64), and colloidal silica (HDK^®^ N20 PHARMA) using a bin blender to obtain a uniform pre-blend. This pre-blend was subsequently granulated using a roller compactor (WP200, Alexanderwerk, Germany). The resulting granules were mixed with D-mannitol (Pearlitol^®^ 200 SD), additional crospovidone, and colloidal silica, followed by lubrication with sucrose esters of fatty acids (DK ESTER F-20W) to yield the final blend. Tablets were compressed into round, biconvex forms (9.0 mm diameter) using a rotary tablet press.

### 2.3. Physical Characterization of Granules, Blends, and Tablets

The physical properties of the granules and final blends were assessed based on particle size distribution, bulk density, and tapped density, while tablet characteristics were evaluated in terms of weight, hardness, and friability. Particle size distribution was determined by sieve analysis using ASTM-standard sieves No. 20 (850 μm), No. 35 (500 μm), No. 60 (250 μm), and No. 100 (150 μm). Approximately 10 g of each sample was loaded onto the top sieve and subjected to vibration for 5 min using a sieve shaker (Retsch AS200). The mass retained on each sieve was recorded and expressed as a percentage of the total sample weight. Bulk density was measured following USP <616> Method I by filling a 100 mL graduated cylinder with granules or blends and recording the weight. Tapped density was determined by tapping the cylinder 1250 times using a tapped density tester (ERWEKA SVM II) in accordance with USP <616> Method I. Tablet weight was measured using an analytical balance (Sartorius), hardness using a tablet hardness tester (ERWEKA TBH 325), and friability using a friability tester (ERWEKA TAR II).

### 2.4. Assessment of Blend Uniformity Using HPLC

To evaluate blend uniformity, a representative sample equivalent to approximately 1–3 times the unit dose was accurately weighed and transferred into a 100 mL volumetric flask. The sample was dissolved in 50% (*v*/*v*) acetonitrile solution with gentle agitation to ensure complete dissolution. A 5 mL aliquot of this solution was then diluted to 50 mL using the same solvent. The resulting solution was filtered through a 0.45 μm membrane filter and transferred into a clean HPLC vial for analysis. Chromatographic analysis was performed using a high-performance liquid chromatography (HPLC) system (Hitachi L-2420, Tokyo, Japan) operated with Empower software (https://www.empower.com/, (accessed on 15 May 2025)). An Inertsil^®^ C18 column (5.0 μm, 150 mm × 4.6 mm, GL Science Inc., Tokyo, Japan) was used as the stationary phase, and the column oven was maintained at 40 °C. The mobile phase consisted of 5 mM phosphate buffer and acetonitrile in a volumetric ratio of 1:10, delivered at a flow rate of 1.0 mL/min. The injection volume was 10 μL, and the analyte was detected at a wavelength of 220 nm. A standard solution was prepared by dissolving 110.69 mg of clopidogrel napadisilate monohydrate in 50% acetonitrile and diluting 5 mL of this solution to 50 mL under identical conditions. The percentage of drug content in the blend sample was determined using the following equation:

DC(%) = (At/As) × (Ws/100 mL) × (5 mL/50 mL) × (100 mL/Wt) × (50 mL/5 mL) × (M/C) × P

where At and As are the peak areas of the sample and standard solutions, respectively; Ws is the weight of the standard; Wt is the weight of the sample; M is the average tablet weight; C is the labeled content of clopidogrel napadisilate monohydrate per tablet; and P represents the purity of the standard material.

### 2.5. Quantitative Determination of Clopidogrel Content

For the assay of clopidogrel content in the final dosage form, a total of 20 tablets were accurately weighed and ground into a fine powder using a mortar and pestle. A portion of this powder, equivalent to 110.69 mg of clopidogrel napadisilate monohydrate (molecular weight = 949.96), was transferred to a 100 mL volumetric flask and dissolved in 50% acetonitrile solution. After complete dissolution, 5 mL of the solution was taken and diluted to 50 mL with the same solvent. The diluted solution was filtered through a 0.45 μm membrane filter, and the clear filtrate was transferred into an HPLC vial. Chromatographic analysis and calculation of drug content were carried out under the same conditions as described in Section 2.4. The percentage of clopidogrel content was calculated using the same formula, with M representing the average tablet weight obtained from the batch.

### 2.6. Evaluation of Content Uniformity in Tablet Dosage Form

Content uniformity was assessed by analyzing individual tablets. A single tablet was placed in a 100 mL volumetric flask and dissolved in 50% acetonitrile solution. After ensuring complete dissolution, a 5 mL portion was taken and diluted to 50 mL using the same solvent. The resulting solution was filtered through a 0.45 μm membrane filter and placed in an HPLC vial. HPLC analysis was conducted as previously described. The drug content of each tablet was calculated using the same equation, where 1T was used in place of M to reflect individual tablet analysis. This test ensures that each dosage unit contains the intended amount of active pharmaceutical ingredient within the acceptable range as defined by regulatory guidelines.

### 2.7. Drug Release Profiling and Dissolution Testing

The in vitro dissolution profile of clopidogrel tablets was assessed using the USP Apparatus II (paddle method) in accordance with the FDA and Korean Pharmacopoeia guidelines. Each tablet was placed in 900 mL of dissolution medium (pH 2.0) maintained at 37 ± 0.5 °C. The paddle speed was set to 75 rpm. At predefined time points, 5 mL aliquots were withdrawn and replaced with an equal volume of fresh medium to maintain sink conditions. Each collected sample was filtered through a 0.45 μm Millipore membrane filter; the first 1 mL of filtrate was discarded to avoid initial impurities, and the remaining solution was collected in a sample vial for analysis. HPLC conditions, including instrumentation and column specifications, were identical to those described in Section 2.4. The standard solution was prepared by dissolving 110.69 mg of clopidogrel napadisilate monohydrate in 50% acetonitrile and then diluting 5 mL of this solution to 50 mL using the dissolution medium. Drug release at each time point was calculated using the following formula:

DC (%) = (At/As) × (Ws/100 mL) × (5 mL/50 mL) × (900 mL/1T) × (1T/C) × P

where the variables are consistent with those defined in previous sections, and 1T refers to the single tablet analyzed.

## 3. Results

### 3.1. Determination of CQA

As a foundational component of the Quality by Design (QbD) paradigm, the critical quality attributes (CQAs) of the final clopidogrel tablet product were systematically identified through a structured, science- and risk-based approach. This process was designed to establish Proven Acceptable Ranges (PARs) for critical process parameters (CPPs) by clarifying their quantitative and qualitative impact on CQAs across each unit operation of the manufacturing process. A comprehensive risk assessment was conducted to prioritize quality attributes that, if deviated from their specification limits, could significantly compromise the safety, efficacy, or performance of the drug product. Attributes exhibiting high severity were designated as CQAs. Once the CQAs were defined, the interrelationships between unit operation parameters, intermediate critical quality attributes (iCQAs), and final product CQAs were thoroughly evaluated using risk analysis tools. This enabled the identification of CPPs with the greatest influence on product quality, based on severity, occurrence probability, and detectability. For each unit operation, PARs were determined by systematically analyzing the response of iCQAs to varying levels of process parameters. This enabled selection of optimal processing conditions within the defined ranges to ensure robust and reproducible product performance. Mechanistic and statistical understanding of the relationships between CPPs, iCQAs, and CQAs facilitated the implementation of direct control strategies, ensuring real-time assurance of product quality during manufacturing. This integrated approach supports consistent attainment of the predefined Quality Target Product Profile (QTPP), ultimately enhancing process robustness and product reliability. The final list of CQAs for the clopidogrel tablet formulation is presented in Table 3.

A semi-quantitative risk analysis was conducted using a Failure Mode and Effects Analysis (FMEA)-based framework, in alignment with the ICH Q9 guideline. Each critical process parameter (CPP) was assessed by scoring three dimensions—severity (S), occurrence (O), and detectability (D)—on a scale from 1 (lowest risk) to 5 (highest risk). Risk Priority Numbers (RPNs) were calculated as the product of these scores (RPN = S × O × D). Parameters with RPN scores greater than 20 were considered medium or higher risk. This scoring system enabled the identification and prioritization of high-impact CPPs across unit operations in a structured, transparent manner.

### 3.2. Process Parameter Adjustment for Pre-Blending Process

The pre-blending step was a critical unit operation in ensuring sufficient homogeneity of the input materials prior to dry granulation, which in turn contributed to the consistent quality of the resulting granules. This step was particularly important for controlling intermediate critical quality attributes (iCQAs), such as bulk density, tapped density, particle size distribution, and blend uniformity—all of which showed strong correlations with final critical quality attributes (CQAs), including drug content and content uniformity [37]. Key process parameters (CPPs) influencing the iCQAs during pre-blending included the type of blending equipment, the fill ratio of materials, and the number of blending revolutions—factors that were further determined by the blending speed and duration. To systematically identify high-risk parameters, a risk assessment approach was applied based on the severity, occurrence probability, and detectability of their potential impact on CQAs [38].

**Figure 1:**
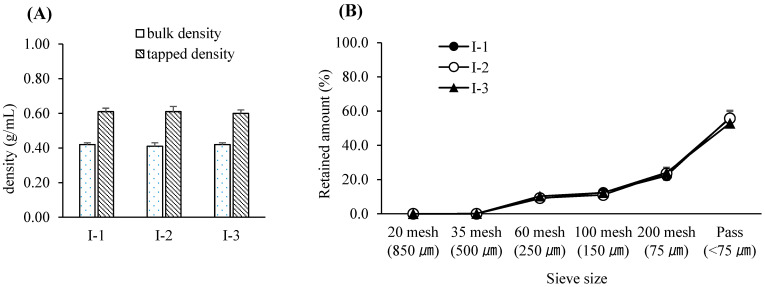
(**A**) Bulk and tapped densities following pre-blending with various blending times. Each value represents the mean ± S.D. (*n* = 3). (**B**) Particle size distribution following pre-blending with various blending times. Each value represents the mean ± S.D. (*n* = 3).

For this study, a bin blender—commonly employed in pharmaceutical manufacturing as a diffusion-type mixing device—was utilized for blending the drug substance with excipients. The working volume of the blender was chosen to maintain an appropriate fill level between 30% and 70%, a range known to ensure sufficient mixing efficiency [39]. The blending speed was fixed at 17 rpm, as determined during equipment qualification, and the number of revolutions was controlled by adjusting the blending time. Among the assessed variables, blending time was identified as the most critical CPP and was selected for further evaluation within a range suitable for typical equipment variability observed in commercial production [40]. To minimize the risk of poor blend uniformity due to agglomeration or material bridging, all input materials were pre-screened using a 1.0 mm sieve prior to blending. Throughout the tested blending time range, the bulk density, tapped density, and particle size distribution of the pre-blend remained consistent, indicating physical stability. Furthermore, the blend uniformity consistently met the predefined specification for drug content—95.0% to 105.0% of the label claim—with each individual unit falling within ±10% of the average and relative standard deviation (RSD) remaining below 5%, thus complying with regulatory and industry acceptance criteria [41]. Based on these results, the Proven Acceptable Range (PAR) for blending time was established as 9–13 min, with a target value set at 11 min. A univariate design was employed at this stage, using predefined blending times to represent practical operating ranges. Full multifactorial DoE was not conducted due to equipment and feasibility constraints. The experimental design for the pre-blending process is summarized in Table 4. The variations in bulk and tapped densities according to blending duration are illustrated in Figure 1A, while corresponding particle size distribution data are presented in Figure 1B. Blend uniformity results for each condition are provided in Table 5.

### 3.3. Optimization of the Dry Granulation Process

The dry granulation step was optimized to improve the flowability and compactability of the formulation by compacting and milling the pre-blended mixture. Granule strength, a factor directly linked to the CQA of drug release, was primarily governed by the compaction pressure applied during roller granulation [42]. A roller compactor was employed to perform this step, and the resulting granules were characterized by iCQAs such as bulk density, tapped density, and particle size distribution. These parameters were found to influence subsequent process performance metrics, including blend uniformity, tabletability, and CQAs such as drug content, content uniformity, and dissolution behavior [43]. Key process variables affecting the iCQAs during roller compaction included roll type, hopper stirrer speed, feeder screw speed, roller speed, roller pressure, roller gap, milling screen size, and milling speed. CPPs were selected using a risk-based approach that considered severity, occurrence probability, and detectability in relation to their potential impact on CQAs [44].

A knurled-type roll was selected based on the non-sticky nature of the formulation, ensuring effective powder compaction. Hopper stirrer speed, although not a major contributor to product variability, was set at a constant value based on prior equipment knowledge, as it primarily functions to support uniform feeding to the screw. Among all variables, roller pressure, roller speed, and roller gap exhibited the most significant influence on iCQAs [45]. Feeder screw speed was automatically adjusted by the equipment to maintain a consistent roller gap, while the milling screen size and milling speed demonstrated minimal variability and were thus considered stable parameters. To assess the criticality of the selected CPPs, a three-factor, two-level factorial design with three center points was employed. The evaluated range was chosen to encompass process variability typically encountered in commercial-scale production. Granules produced under each condition were subjected to downstream post-blending and lubrication, followed by compression to evaluate blend uniformity, tablet hardness, friability, dissolution rate, and content uniformity under varying compression forces. No significant differences were observed in bulk and tapped densities across the conditions. However, notable variations in particle size distribution were detected as a function of the CPP settings. Despite these differences, all post-blended samples met blend uniformity acceptance criteria, with drug content ranging between 95.0 and 105.0% of the label claim, individual values within ±10% of the average, and RSD values under 5%. Tablet content uniformity after compression complied with regulatory limits, not exceeding 15.0% variation per unit [46]. All granules exhibited a consistent increase in tablet strength with rising compression force (5–23 kN), yielding predictable hardness profiles. Friability decreased with increased compression force, remaining below 0.5% across all conditions. Moreover, tablets compressed at low (5 kN), intermediate (14 kN), and high (23 kN) forces met the CQA specification for drug release, demonstrating ≥80% release within 30 min, irrespective of granule properties [47].

**Figure 2:**
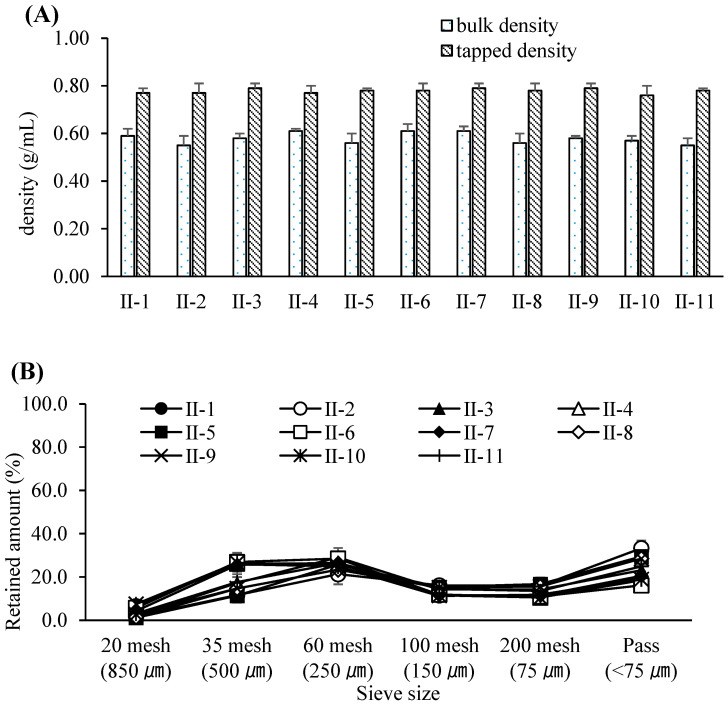
(**A**) Bulk and tapped densities of the compacted granules resulting from various roller compaction process parameters. Each value represents the mean ± S.D. (*n* = 3). (**B**) Particle size distribution of the compacted granules resulting from various roller compaction process parameters. Each value represents the mean ± S.D. (*n* = 3).

**Figure 3:**
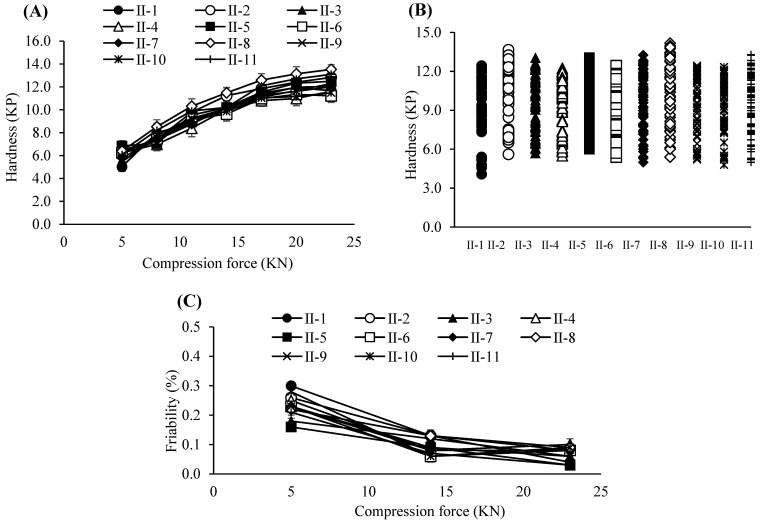
(**A**) Tablet hardness profiles resulting from various compression force and roller compaction process parameters. Each value represents the mean ± S.D. (*n* = 10). (**B**) Tablet hardness plots specified by compacted granules produced by various roller compaction process parameters. (**C**) Tablet friability profiles resulting from various compression force and roller compaction process parameters. Each value represents the mean ± S.D. (*n* = 3).

**Figure 4:**
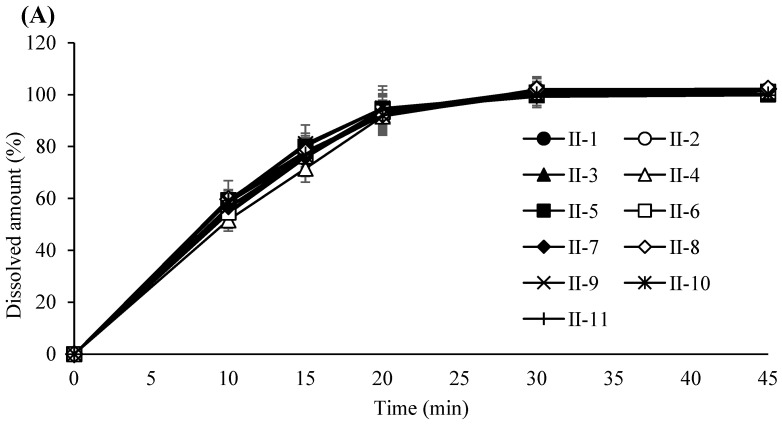

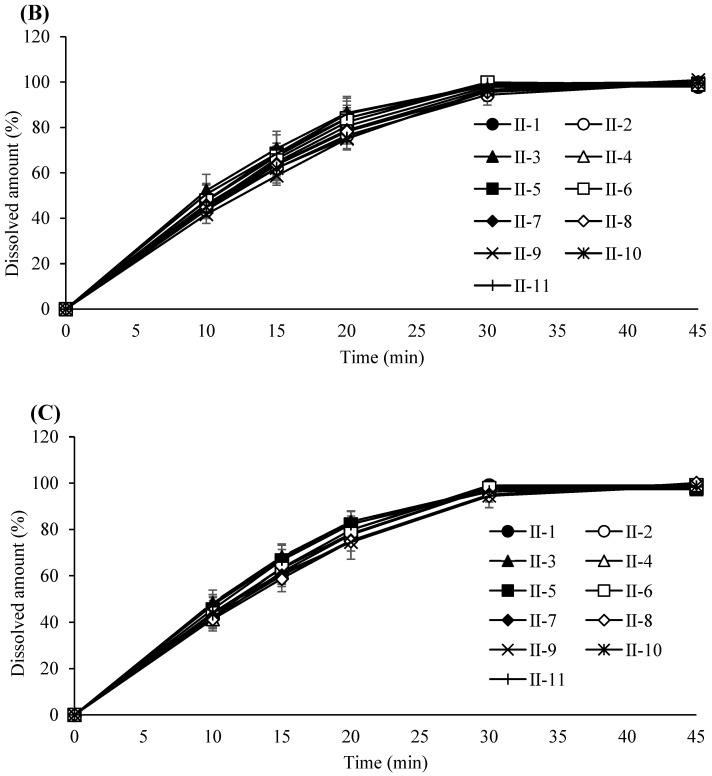
(**A**) Dissolution profile of tablets compressed at a 5 KN compression force. Each value represents the mean ± S.D. (*n* = 6).; (**B**) Dissolution profile of tablets compressed at a 14 KN compression force. Each value represents the mean ± S.D. (*n* = 6). (**C**) Dissolution profile of tablets compressed at a 23 KN compression force. Each value represents the mean ± S.D. (*n* = 6).

Based on these results, the PARs for dry granulation were established as follows: roller pressure, 7–9 MPa; roller speed, 20–30 rpm; and roller gap, 2.0–3.0 mm. Acceptance criteria for granule and tablet quality attributes were set at 0.55–0.61 g/mL for granule bulk density, 0.76–0.79 g/mL for tapped density, and 6–12 kP for tablet hardness. The experimental design of the dry granulation process is summarized in Table 6. Bulk and tapped densities under varying roller conditions are shown in Figure 2A, and particle size distribution profiles in Figure 2B. Blend and tablet content uniformity data appear in Table 7. Figure 3A–C illustrate tablet hardness and friability results across compression forces and granulation conditions. Dissolution profiles at three compression forces (5, 14, and 23 kN) are depicted in Figure 4A–C.

Among all evaluated attributes, four response variables—blend uniformity (as RSD), content uniformity (as acceptance value), bulk density, and tapped density—were selected for statistical modeling. These variables were uniformly measured across all batches and correspond directly to the controlled process factors in the DoE. Granule size (D50), hardness, friability, and dissolution profiles were excluded due to dependence on additional test conditions (e.g., variable compression forces or timepoints), or due to limited numeric batch-level data. All statistical analyses, including regression modeling and ANOVA, were performed using Minitab^®^ Statistical Software, version 21.4.2 (Minitab LLC, State College, PA, USA). Detailed statistical outputs including regression equations, ANOVA tables, and model adequacy results are provided in Supplementary Tables S1–S3.

### 3.4. Evaluation and Refinement of Post-Blending Conditions

The post-blending step was conducted to ensure adequate homogeneity of the granule–excipient mixture, which is critical for achieving content uniformity in the final tablet product [37]. At this stage, intermediate critical quality attributes (iCQAs)—including bulk density, tapped density, particle size distribution, and blend uniformity—were carefully monitored due to their direct influence on final CQAs such as drug content and its uniformity. Process variables affecting these iCQAs included the type of blending equipment, fill ratio of input materials, and the number of blending revolutions [48], which were themselves governed by blending speed and duration. Critical process parameters (CPPs) were identified through a structured risk assessment based on severity, occurrence probability, and detectability of their potential impact on CQAs.

A conventional diffusion-based blending method was employed using a bin blender. Both granules and extra-granular excipients were pre-screened through a 1.0 mm sieve to prevent agglomeration prior to blending. The same model of bin blender used during the pre-blending step was utilized to maintain equipment consistency. The fill ratio of materials was kept between 30% and 70%, a range considered optimal for achieving adequate mixing dynamics in industrial-scale blenders. Blending speed was fixed at 17 rpm as qualified during equipment validation, while blending time was varied to adjust the total number of revolutions. Blending time was identified as the most critical parameter and was therefore selected as the CPP for optimization. Its evaluation range was defined to encompass variability encountered during routine campaign production. Across all tested blending durations, no significant changes were observed in bulk density, tapped density, or particle size distribution of the final post-blend. Furthermore, blend uniformity consistently met the predefined CQA specification for drug content—ranging from 95.0% to 105.0% of the label claim—with individual sample RSD values under 5% and all individual measurements falling within ±10% of the average, thereby meeting industry acceptance standards [46].

**Figure 5:**
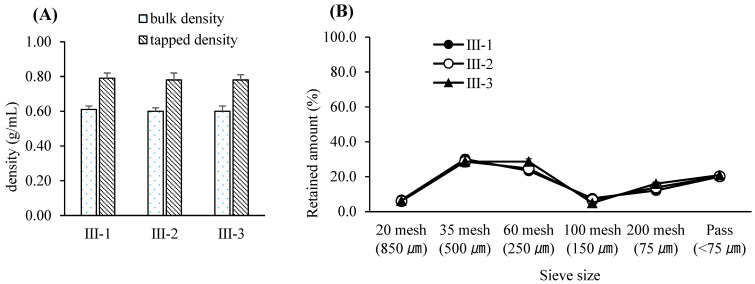
(**A**) Bulk and tapped densities of post-blended granules resulting from various blending times. Each value represents the mean ± S.D. (*n* = 3). (**B**) Particle size distribution of post-blended granules resulting from various blending times. Each value represents the mean ± S.D. (*n* = 3).

Based on the results, the Proven Acceptable Range (PAR) for blending time in the post-blending step was determined to be 9–13 min, with a target blending time of 11 min [49]. As in the pre-blending step, a univariate approach was applied rather than a factorial design, with blending times selected to reflect industry-standard operation windows. The design parameters for the post-blending process are summarized in Table 8. The resulting bulk and tapped densities at various blending times are illustrated in Figure 5A, while particle size distributions are shown in Figure 5B. Blend uniformity data corresponding to each blending condition are presented in Table 9.

### 3.5. Impact of Lubrication Parameters on Tablet Performance

The lubrication step was conducted to incorporate a lubricant into the homogenous post-blend in order to prevent powder adhesion to the tablet punches during compression and to ensure uniform tablet appearance. However, excessive lubrication is known to adversely affect the dissolution rate of tablets, emphasizing the importance of precise process control during this step [50]. Intermediate critical quality attributes (iCQAs) of the final blend—specifically bulk density, tapped density, particle size distribution, and tabletability—were monitored due to their strong association with CQAs such as drug release and tablet appearance. Process variables influencing these iCQAs included the type of lubrication equipment, fill ratio of input materials, and number of lubrication revolutions, which were determined by lubrication speed and time. Critical process parameters (CPPs) were selected through risk assessment by evaluating the severity, probability, and detectability of their impact on CQAs [51]. The same bin blender used in the pre-blending and post-blending steps was employed for the lubrication process to maintain consistency in equipment qualification. The fill level was maintained between 30% and 70% to ensure effective blending performance. The lubrication speed was fixed at 17 rpm, as qualified during equipment validation, while lubrication time was varied to control the number of revolutions. Among the evaluated variables, lubrication time was identified as the most critical and was therefore selected as the CPP for optimization. It was assessed over a range capable of accommodating equipment variability typically encountered during commercial-scale production. Across the tested lubrication durations, no significant changes were observed in bulk density, tapped density, or particle size distribution of the final blend. For each condition, the lubricated blend was sampled and compressed into tablets at compression forces ranging from 5 to 23 kN to evaluate tabletability and dissolution behavior. No tableting defects—such as sticking, capping, or edge erosion—were noted during compression [52]. Furthermore, no significant differences were observed in tablet hardness profiles across the tested conditions. The dissolution rate of tablets compressed at the highest force (23 kN) consistently met the CQA requirement of not less than 80% drug release within 30 min, indicating that the level of lubrication did not hinder dissolution performance [47]. Based on these findings, the Proven Acceptable Range (PAR) for lubrication time was established as 3–5 min, with a target value of 4 min. This evaluation was based on fixed-level testing rather than factorial design. Although this limits interaction analysis, the selected levels reflect commonly validated ranges in industrial manufacturing.

**Figure 6:**
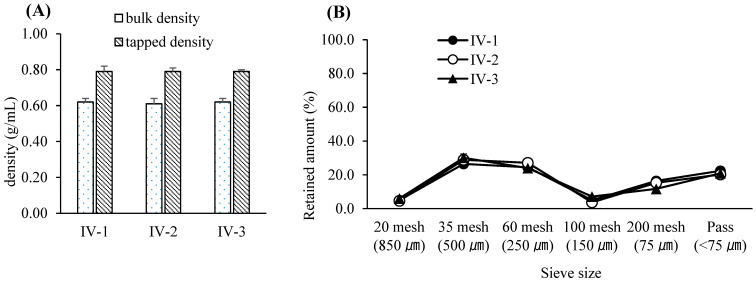
(**A**) Bulk and tapped densities of final-blend granules resulting from various lubrication times. Each value represents the mean ± S.D. (*n* = 3). (**B**) Particle size distribution of final-blended granules resulting from various lubrication times. Each value represents the mean ± S.D. (*n* = 3).

**Figure 7:**
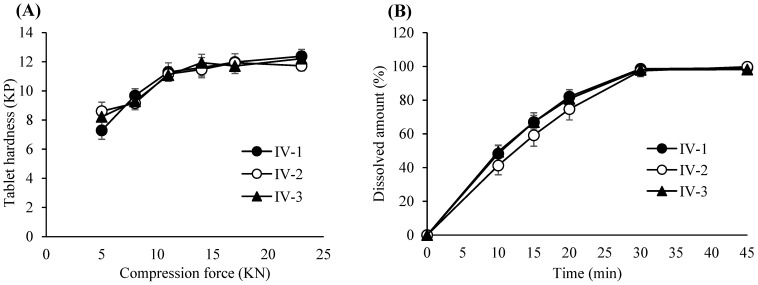
(**A**) Tablet hardness profiles resulting from different compression forces and lubrication times. Each value represents the mean ± S.D. (*n* = 10). (**B**) Dissolution profiles of the tablets compressed at a 23 KN compression force and different lubrication times. Each value represents the mean ± S.D. (*n* = 10).

The design of the lubrication process is summarized in Table 10. Figure 6A presents the bulk and tapped densities of the final blend under different lubrication durations, while Figure 6B illustrates the corresponding particle size distributions. Tablet hardness profiles obtained from varying compression forces and lubrication times are shown in Figure 7A, and dissolution profiles of tablets compressed at 23 kN under different lubrication conditions are depicted in Figure 7B.

### 3.6. Compression Process Control for Tablet Robustness

The tablet compression step was performed to transform the final lubricated blend into tablets with acceptable appearance, uniform drug content, and the intended drug release characteristics [53]. Key tablet quality attributes included average weight, hardness, and friability, each of which was linked to one or more critical quality attributes (CQAs) such as appearance, content uniformity, and dissolution performance. Among these, tablet average weight and its variability were considered intermediate critical quality attributes (iCQAs) due to their direct relationship with drug content and content uniformity [54]. Tablet hardness was also defined as an iCQA, as it reflects mechanical strength and influences disintegration and dissolution. In contrast, friability was deemed a non-critical attribute [55], given its indirect association with CQAs through its correlation with hardness. Process variables affecting these iCQAs included the type of compression equipment, punch shape, turret speed, feeder speed, filling depth, and compression force. Critical process parameters (CPPs) were identified through risk assessment by evaluating the severity, probability, and detectability of each parameter’s potential impact on CQAs. A conventional rotary tablet press equipped with 36 punch stations was utilized. Among the assessed variables, turret speed and feeder speed were found to have the greatest impact on average tablet weight and its uniformity. Filling depth and compression force could be controlled based on in-process monitoring of tablet weight and hardness and were thus considered lower-risk variables. The punch shape, which was uniformly round in this study, also posed minimal variability. Consequently, turret speed and feeder speed were selected as CPPs and evaluated across a range that encompassed typical operating conditions in commercial manufacturing.

To assess the effect of these parameters, tablets were produced using various combinations of turret and feeder speeds. The compression force was adjusted to consistently achieve a target hardness of 10 kiloponds (kP). The resulting tablets were evaluated for average weight, assay, individual weight variation, content uniformity, friability, and dissolution. Across all tested conditions, average tablet weight met the specification of 305 mg ± 3%, and assay values conformed to the CQA requirement of 95.0–105.0% of the labeled drug content. Individual weight variation remained within ±5% of the average value, and the content uniformity acceptance value did not exceed the regulatory limit of 15.0%. Friability remained below 0.5% under all conditions, and the dissolution rate exceeded 80% drug release within 30 min, satisfying the CQA criteria for drug release.

**Figure 8:**
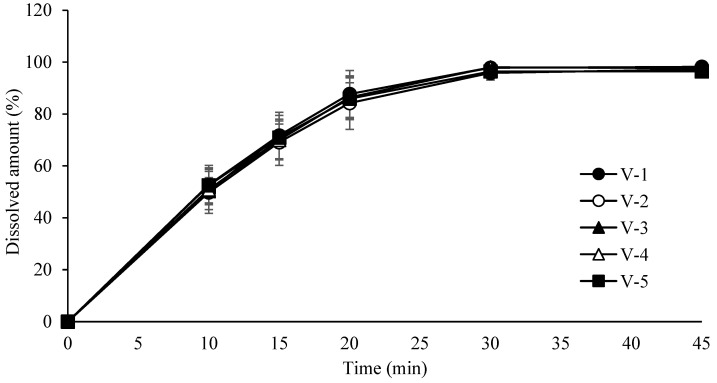
Dissolution profiles of tablets compressed at different turret and feeder speeds. Each value represents the mean ± S.D. (*n* = 6).

Based on these results, the Proven Acceptable Ranges (PARs) were established as 15–35 rpm for turret speed (target value: 25 rpm) and 60–100 rpm for feeder speed (target value: 80 rpm). Due to the limited number of controllable CPPs at this stage, full multifactorial DoE was not performed. Instead, a univariate assessment of turret and feeder speeds was used to identify acceptable performance windows. The design of the tablet compression process is summarized in Table 11. Evaluation results for weight, assay, content uniformity, hardness, and friability are provided in Table 12. Dissolution profiles for tablets produced under different turret and feeder speed settings are shown in Figure 8.

## 4. Conclusions

The clopidogrel tablet manufacturing process was successfully optimized using the Quality by Design (QbD) framework. Through systematic identification and evaluation of critical process parameters (CPPs) and their associated intermediate critical quality attributes (iCQAs), the process parameters impacting critical quality attributes (CQAs) were thoroughly characterized. Proven Acceptable Ranges (PARs) were established for each CPP, forming the foundation for a comprehensive control strategy aimed at ensuring consistent product quality. This QbD-based approach significantly enhanced process understanding by integrating scientific rationale and risk-based decision-making. The resulting control strategy supports robust manufacturing of clopidogrel tablets that consistently meet target quality specifications. Moreover, the framework allows for greater operational flexibility, enabling proactive management of process variability and reducing the risk of batch failure, reprocessing, or unnecessary product rejection. Overall, the application of QbD not only improves quality assurance but also contributes to cost-effective production. In particular, this study advances the field by establishing direct linkages between dry granulation-specific CPPs and CQAs for clopidogrel, offering a new reference model for high-risk BCS class II drugs. The final control strategy for the clopidogrel tablet manufacturing process is summarized in Table 13.

## Figures and Tables

**Table 1:** Summary of QbD-integrated studies.

Application Area	QbD Concept Applied	Key Emphasis of QbD	Reference
Amorphous solid dispersion (ASD) formulation development	Optimization of process parameters and enhancement of dissolution performance	Improved solubility and stability of the final formulation	[11]
Solid dispersion of sorafenib tosylate (SFN)	Design of experiment (DoE)-based optimization for enhanced pharmacokinetics	Over 20-fold improvement in solubility and oral bioavailability	[24]
Liposomal nano-drug delivery systems	Systematic experimental design and risk assessment	Enhanced reproducibility and efficiency in complex nanoformulation development	[25]
mRNA vaccine development	Establishment of standardized quality control protocols	Improved process control and impurity profiling for high-quality mRNA vaccines	[26]
Self-amplifying mRNA (saRNA) vaccines	Optimization of stability, immunogenicity, and safety	Enhanced antigen expression and immune modulation	[27]
Nanoformulation-based drug delivery systems (NDDSs)	Optimization of critical parameters such as ZP ^3^, particle size, EE ^4^, and PDI ^5^	Improved quality and reliability in nanoparticle manufacturing	[28]
Nanoparticle liposomes for anticancer therapy	Evaluation of formulation variables including drug–lipid ratio and processing conditions	Achievement of predefined quality attributes for enhanced therapeutic performance	[29]
Ophthalmic delivery using SLN ^1^, NLC ^2^, and nanoemulsion systems	D-optimal mixture design and physicochemical characterization	Anti-inflammatory efficacy and robust formulation development	[30]

^1^ SLN solid lipid nanoparticles, ^2^ NLC nanostructured lipid carriers, ^3^ ZP zeta potential, ^4^ EE entrapment efficiency, ^5^ PDI polydispersity index.

**Table 2:** Composition of clopidogrel-loaded tablets.

Manufacturing Process	Ingredients	mg/T
Pre-blending and roller compaction	Clopidogel napadisilate—loaded solid dispersion	171.2 ^1^
	Fumaric acid	35.0
	Crospovidone	25.0
	Copovidone	5.0
	Colloidal silica	5.0
Post-blending	D-mannitol	31.8
	Crospovidone	10.0
	Colloidal silica	12.5
Lubrication	Sucrose esters of fatty acids	9.5
Total weight	305.0	

^1^ Equivalent to 75 mg clopidogrel.

**Table 3:** Critical quality attributes (CQAs) of clopidogrel tablets.

Critical Quality Attribute (CQA)	Specification
Appearance	Off-white round tablet
Identification	Retention time of standard solution and test solution is the same
Drug content	95.0–105.0% of label claim
Uniformity of drug content	Acceptance value within 15.0%
Drug release	Not less than 80.0% in 30 min

**Table 4:** Study design of the pre-blending process.

Batch	I-1	I-2	I-3
Blending speed (rpm)	17	17	17
Blending time (min)	9	11	13
Blending revolution number (rev.) *	153	187	221

* Blending speed × blending time.

**Table 5:** Blend uniformity following pre-blending with various blending times.

Batch		I-1	I-2	I-3
Blend uniformity (10 locations, %)	Average	99.3	99.4	98.7
	RSD	1.6	1.3	1.2
	Min	98.0	98.1	97.7
	Max	101.2	100.5	99.9

**Table 6:** Study design of the dry granulation process.

Batch	II-1	II-2	II-3	II-4	II-5	II-6	II-7	II-8	II-9	II-10	II-11
Roller pressure (MPa)	9	9	8	9	7	9	8	7	7	7	8
Roller speed (rpm)	20	30	25	20	20	30	25	30	20	30	25
Roller gap (mm)	3.0	3.0	2.5	2.0	3.0	2.0	2.5	3.0	2.0	2.0	2.5

**Table 7:** Blend and tablet content uniformities of the final blend of compacted granules resulting from various roller compaction process parameters.

Batch		II-1	II-2	II-3	II-4	II-5	II-6	II-7	II-8	II-9	II-10	II-11
Blend uniformity (10 locations, %)	Average	101.0	99.8	99.7	99.8	101.0	100.4	99.8	101.0	101.3	100.2	100.1
	RSD	1.0	1.2	1.8	1.7	1.4	0.8	0.7	1.1	0.9	0.8	0.9
	Min	99.9	97.0	96.8	96.9	98.4	99.0	98.5	99.0	100.2	98.6	98.8
	Max	103.2	101.2	102.5	101.8	102.9	101.3	100.7	102.2	102.8	101.0	101.2
Content uniformity (10 tablets, %)	Average	101.2	102.0	101.6	101.0	99.5	102.5	102.5	103.1	100.9	100.5	101.5
	SD	0.5	1.0	0.6	0.7	0.5	0.9	0.7	1.0	0.9	1.3	0.7
	Acceptance value	1.2	2.9	1.5	1.7	1.2	3.2	2.7	4.0	2.2	3.1	1.7

**Table 8:** Study design of the post-blending process.

Batch	III-1	III-2	III-3
Blending speed (rpm)	17	17	17
Blending time (min)	9	11	13
Blending revolution number (rev.) *	153	187	221

* Blending speed × blending time.

**Table 9:** Blend uniformity of post-blended granules resulting from various blending times.

Batch		I-1	I-2	I-3
Blend uniformity (10 locations, %)	Average	99.8	99.4	99.1
	RSD	1.2	0.7	0.8
	Min	98.0	98.1	97.7
	Max	101.2	100.5	99.9

**Table 10:** Study design of the lubrication process.

Batch	IV-1	IV-2	IV-3
Lubrication speed (rpm)	17	17	17
Lubrication time (min)	3	4	5
Lubrication revolution number (rev.) *	51	68	85

* Lubrication speed × lubrication time.

**Table 11:** Study design of the tablet compression process.

Batch	V-1	V-2	V-3	V-4	V-5
Turret speed (rpm)	15	25	35	15	35
Feeder speed (rpm)	60	80	100	100	60

**Table 12:** Result of the tablet compression process. Each value represents the mean ± S.D.

Batch	V-1	V-2	V-3	V-4	V-5
Tablet weight (*n* = 20, mg)	303.3 ±2.7	305.1 ±3.7	304.7 ±3.7	307.5 ±2.1	305.3 ±4.1
Assay (*n* = 3, %)	99.9 ±0.5	100.0 ±0.3	98.6 ±0.3	99.4 ±0.2	98.5 ±0.4
Content uniformity (*n* = 10, %)	100.5 ±1.7	99.5 ±1.5	99.4 ±1.2	99.2 ±1.7	98.9 ±1.0
Content uniformity acceptance value (%)	4.1	3.6	2.9	4.1	4.1
Tablet hardness (*n* = 10, KP)	9.7 ±1.0	9.9 ±1.3	10.0 ±1.4	9.8 ±0.9	10.4 ±1.4
Tablet friability (*n* = 3, %)	0.18 ±0.01	0.19 ±0.02	0.20 ±0.01	0.20 ±0.01	0.18 ±0.02

**Table 13:** Control strategy of clopidogrel tablet manufacturing process.

Process Step	Critical Process Parameter (CPP)	Proven Acceptance Range (PAR)	Set Point
Pre-blending	Blending time	9–13 min	11 min
Roller compaction	Roller speed	20–30 rpm	25 rpm
	Roller pressure	7–9 MPa	8 MPa
	Roller gap	2.0–3.0 mm	2.5 mm
Post-blending	Blending time	9–13 min	11 min
Lubrication	Lubrication time	3–5 min	4 min
Compression	Turret speed	15–35 rpm	25 rpm
	Feeder speed	60–100 rpm	80 rpm

## Data Availability

Data are available on request due to restrictions, e.g., privacy or ethical restrictions.

## Supplementary Materials

The following supporting information can be downloaded at https://www.mdpi.com/article/10.3390/pharmaceutics17050659/s1. Table S1. Regression Equations and R^2^ Values; Table S2. ANOVA Results for Dry Granulation Factors; Table S3. Model Adequacy Tests.

## References

[1] Grangeia H.B., Silva C., Simões S.P., Reis M.S. (2020). Quality by design in pharmaceutical manufacturing: A systematic review of current status, challenges and future perspectives. Eur. J. Pharm. Biopharm..

[2] Yu L.X., Amidon G., Khan M.A., Hoag S.W., Poli J., Raju G.K., Woodcock J. (2014). Understanding pharmaceutical Quality by Design. AAPS J..

[3] ICH (2009). International Conference on Harmonisation of Technical Requirements for Registration of Pharmaceuticals for Human Use, Pharmaceutical Development Q8(R2).

[4] ICH (2008). International Conference on Harmonisation of Technical Requirements for Registration of Pharmaceuticals for Human Use, Pharmaceutical Quality System Q10.

[5] Swain S., Parhi R., Jena B.R., Babu S.M. (2019). Quality by Design: Concept to applications. Curr. Drug Discov. Technol..

[6] Namjoshi S., Dabbaghi M., Roberts M.S., Grice J.E., Mohammed Y. (2020). Quality by Design: Development of the Quality Target Product Profile (QTPP) for semisolid topical products. Pharmaceutics.

[7] Mohseni-Motlagh S.F., Dolatabadi R., Baniassadi M., Baghani M. (2023). Application of the Quality by Design concept (QbD) in the development of hydrogel-based drug delivery systems. Polymers.

[8] Teng K., Fu H., Wu G., Gong P., Xie Y., Zhou P., Gong X., Qu H. (2023). QbD-guided traditional Chinese medicine manufacturing process: Development and optimization of fluid-bed granulation and drying processes for Xiaochaihu capsules. AAPS PharmSciTech.

[9] Zhang H., Wang B., Liu X., Zhang H., Yao J., Gong X., Yan J. (2022). Process optimization for the synthesis of functionalized Au@AgNPs for specific detection of Hg^2+^ based on Quality by Design (QbD). RSC Adv..

[10] Rampado R., Peer D. (2023). Design of experiments in the optimization of nanoparticle-based drug delivery systems. J. Control. Release.

[11] Shukla A., Dumpa N.R., Thakkar R., Shettar A., Ashour E., Bandari S., Repka M.A. (2023). Influence of Poloxamer on the dissolution and stability of hot-melt extrusion-based amorphous solid dispersions using design of experiments. AAPS PharmSciTech.

[12] Ter Horst J.P., Turimella S.L., Metsers F., Zwiers A. (2021). Implementation of Quality by Design (QbD) principles in regulatory dossiers of medicinal products in the European Union (EU) between 2014 and 2019. Ther. Innov. Regul. Sci..

[13] Zagalo D.M., Sousa J., Simões S. (2022). Quality by Design (QbD) approach in marketing authorization procedures of non-biological complex drugs: A critical evaluation. Eur. J. Pharm. Biopharm..

[14] Singh S., Chaurasia A., Gupta N., Rajput D.S. (2024). Effect of formulation parameters on enalapril maleate mucoadhesive buccal tablet using Quality by Design (QbD) approach. Zhongguo Ying Yong Sheng Li Xue Za Zhi.

[15] Gurba-Bryśkiewicz L., Maruszak W., Smuga D.A., Dubiel K., Wieczorek M. (2023). Quality by Design (QbD) and design of experiments (DOE) as a strategy for tuning lipid nanoparticle formulations for RNA delivery. Biomedicines.

[16] Osmanović Omerdić E., Alagić-Džambić L., Krstić M., Pašić-Kulenović M., Medarević Đ., Ivković B., Vasiljević D. (2022). Long-term stability of clopidogrel solid dispersions—Importance of in vitro dissolution test. PLoS ONE.

[17] Singh A., Sharma R., Chaudhary S., Rana V. (2023). Preparation and characterization of clopidogrel bisulfate-hydroxypropyl-β-cyclodextrin mixed inclusion complex for improved intestinal solubility and anti-thrombotic efficacy. J. Pharm. Sci..

[18] Correa Soto C.E., Gao Y., Indulkar A.S., Ueda K., Zhang G.G.Z., Taylor L.S. (2022). Impact of surfactants on the performance of clopidogrel-copovidone amorphous solid dispersions: Increased drug loading and stabilization of nanodroplets. Pharm. Res..

[19] Shekhawat P., Pokharkar V. (2019). Risk assessment and QbD based optimization of an Eprosartan mesylate nanosuspension: In-vitro characterization, PAMPA and in-vivo assessment. Int. J. Pharm..

[20] Oh G.H., Kim J.E., Park Y.J. (2018). Development of stabilized tenofovir disoproxil tablet: Degradation profile, stabilization, and bioequivalence in beagle dogs. Drug Dev. Ind. Pharm..

[21] Reddy J.P., Phanse R., Nesarikar V. (2019). Parameter estimation for roller compaction process using an instrumented Vector TF mini roller compactor. Pharm. Dev. Technol..

[22] Guo Y., Martinez L., Palanisamy A., Gururajan B., Sun C.C. (2024). An evaluation of six techniques for measuring porosity of ribbons produced by roller compaction. Int. J. Pharm..

[23] Wang C., Wang Z., Friedrich A., Sun C.C. (2022). Effect of deaeration on processability of poorly flowing powders by roller compaction. Int. J. Pharm..

[24] Panda B.K., Chellampillai B., Ghodake S., Mali A.J., Kamble R. (2024). Investigation of magnesium aluminometasilicate (Neusilin US2) based surface solid dispersion of sorafenib tosylate using QbD approach: In vitro and in vivo pharmacokinetic study. ADMET DMPK.

[25] Alshaer W., Nsairat H., Lafi Z., Hourani O.M., Al-Kadash A., Esawi E., Alkilany A.M. (2022). Quality by Design approach in liposomal formulations: Robust product development. Molecules.

[26] Hu C., Bai Y., Liu J., Wang Y., He Q., Zhang X., Cheng F., Xu M., Mao Q., Liang Z. (2024). Research progress on the quality control of mRNA vaccines. Expert Rev. Vaccines.

[27] Pourseif M.M., Masoudi-Sobhanzadeh Y., Azari E., Parvizpour S., Barar J., Ansari R., Omidi Y. (2022). Self-amplifying mRNA vaccines: Mode of action, design, development and optimization. Drug Discov. Today.

[28] Birla D., Khandale N., Bashir B., ShahbazAlam M., Vishwas S., Gupta G., Dureja H., Kumbhar P.S., Disouza J., Patravale V. (2025). Application of quality by design in optimization of nanoformulations: Principle, perspectives and practices. Drug Deliv. Transl. Res..

[29] Kapoor D., Sharma S., Verma K., Bisht A., Sharma M., Singhai N.J., Raval N., Maheshwari R. (2022). Quality-by-design-based engineered liposomal nanomedicines to treat cancer: An in-depth analysis. Nanomedicine.

[30] Uner B., Ozdemir S., Tas C., Uner M., Ozsoy Y. (2023). Loteprednol-loaded nanoformulations for corneal delivery by Quality-by-Design concepts: Optimization, characterization, and anti-inflammatory activity. AAPS PharmSciTech.

[31] Ralbovsky N.M., Smith J.P. (2023). Process analytical technology and its recent applications for asymmetric synthesis. Talanta.

[32] Zhong L., Gao L., Li L., Zang H. (2020). Trends—Process analytical technology in solid oral dosage manufacturing. Eur. J. Pharm. Biopharm..

[33] Su Q., Bommireddy Y., Shah Y., Ganesh S., Moreno M., Liu J., Gonzalez M., Yazdanpanah N., O’Connor T., Reklaitis G.V. (2019). Data reconciliation in the Quality-by-Design (QbD) implementation of pharmaceutical continuous tablet manufacturing. Int. J. Pharm..

[34] Vo A.Q., Kutz G., He H., Narala S., Bandari S., Repka M.A. (2020). Continuous manufacturing of ketoprofen delayed-release pellets using melt extrusion technology: Application of QbD design space, inline near infrared, and inline pellet size analysis. J. Pharm. Sci..

[35] Fiedler D., Fink E., Aigner I., Leitinger G., Keller W., Roblegg E., Khinast J.G. (2023). A multi-step machine learning approach for accelerating QbD-based process development of protein spray drying. Int. J. Pharm..

[36] Munir N., Nugent M., Whitaker D., McAfee M. (2021). Machine learning for process monitoring and control of hot-melt extrusion: Current state of the art and future directions. Pharmaceutics.

[37] Jakubowska E., Ciepluch N. (2021). Blend segregation in tablets manufacturing and its effect on drug content uniformity—A review. Pharmaceutics.

[38] Pathodiya M. (2024). Cross-contamination risk assessment using FMEA tool. J. Drug Deliv. Ther..

[39] Remy B., Glasser B.J., Khinast J.G. (2009). The effect of mixer properties and fill level on granular flow in a bladed mixer. AIChE J..

[40] Razavi S.M., Scicolone J., Snee R.D., Kumar A., Bertels J., Cappuyns P., Van Assche I., Cuitiño A.M., Muzzio F. (2020). Prediction of tablet weight variability in continuous manufacturing. Int. J. Pharm..

[41] Goodwin D.J., van den Ban S., Denham M., Barylski I. (2018). Real time release testing of tablet content and content uniformity. Int. J. Pharm..

[42] Arndt O.-R., Baggio R., Adam A.K., Harting J., Franceschinis E., Kleinebudde P. (2018). Impact of different dry and wet granulation techniques on granule and tablet properties: A comparative study. J. Pharm. Sci..

[43] Han J.K., Shin B.S., Choi D.H. (2019). Comprehensive study of intermediate and critical quality attributes for process control of high-shear wet granulation using multivariate analysis and the Quality by Design approach. Pharmaceutics.

[44] Borchert D., Zahel T., Thomassen Y.E., Herwig C., Suarez-Zuluaga D.A. (2019). Quantitative CPP evaluation from risk assessment using integrated process modeling. Bioengineering.

[45] Atanaskova E., Kostovski D., Anevska-Stojanovska N. (2020). Investigation of the influence of critical process parameters in roller compaction process on physical properties of granules and tablets using design of experiments. Arh. Farm..

[46] Hudson-Curtis B., Novick S. (2016). Assessing content uniformity. Nonclinical Statistics for Pharmaceutical and Biotechnology Industries.

[47] Patel S., Kaushal A.M., Bansal A.K. (2006). Compression physics in the formulation development of tablets. Crit. Rev. Ther. Drug Carrier Syst..

[48] Alyami H., Dahmash E., Bowen J., Mohammed A.R. (2017). An investigation into the effects of excipient particle size, blending techniques and processing parameters on the homogeneity and content uniformity of a blend containing low-dose model drug. PLoS ONE.

[49] Bekaert B., Grymonpré W., Novikova A., Vervaet C., Vanhoorne V. (2022). Impact of blend properties and process variables on the blending performance. Int. J. Pharm..

[50] Paul S., Sun C.C. (2018). Systematic evaluation of common lubricants for optimal use in tablet formulation. Eur. J. Pharm. Sci..

[51] Jiwa N., Ozalp Y., Yegen G., Aksu B. (2021). Critical tools in tableting research: Using compaction simulator and Quality by Design (QbD) to evaluate lubricants’ effect in direct compressible formulation. AAPS PharmSciTech.

[52] Matji A., Donato N., Gagol A., Morales E., Carvajal L., Serrano D.R., Worku Z.A., Healy A.M., Torrado J.J. (2019). Predicting the critical quality attributes of ibuprofen tablets via modelling of process parameters for roller compaction and tabletting. Int. J. Pharm..

[53] Van Snick B., Grymonpré W., Dhondt J., Pandelaere K., Di Pretoro G., Remon J.P., De Beer T., Vervaet C., Vanhoorne V. (2018). Impact of blend properties on die filling during tableting. Int. J. Pharm..

[54] Osei-Yeboah F., Sun C.C. (2015). Validation and applications of an expedited tablet friability method. Int. J. Pharm..

[55] Schomberg A.K., Kwade A., Finke J.H. (2020). The challenge of die filling in rotary presses—A systematic study of material properties and process parameters. Pharmaceutics.

