# Supplementary material for: Advanced QbD-Based Process Optimization of Clopidogrel Tablets with Insights into Industrial Manufacturing Design
Source: Pharmaceutics. 2025 May 17;17(5):659. doi: 10.3390/pharmaceutics17050659 (PMC12114868; doi:10.3390/pharmaceutics17050659)
Supplement: Supplementary file 1 [file pharmaceutics-17-00659-s001.zip › pharmaceutics-3612024-supplementary.pdf]

Supplementary Tables – DoE Analysis

Table S1. Regression Equations and R^2 Values

Response	Regression Equation	R^2
Blend Uniformity (RSD)	BU RSD = -0.92 + 0.275*RollerPressure + 0.0031*RollerSpeed - 0.103*RollerGap	0.52
Content Uniformity (AV)	CU AV = 6.30 - 0.637*RollerPressure - 0.0134*RollerSpeed + 0.599*RollerGap	0.544
Bulk Density	Bulk Density = 0.712 - 0.00718*RollerPressure - 0.00227*RollerSpeed - 0.0071*RollerGap	0.236
Tapped Density	Tapped Density = 0.7767 - 0.00360*RollerPressure + 0.001167*RollerSpeed + 0.00056*RollerGap	0.445

Note: Blend Uniformity and Content Uniformity were analyzed using RSD and Acceptance Value (AV), respectively. Although the R^2 values vary, all models are included for completeness and transparency.

Table S2. ANOVA Results for Dry Granulation Factors

Factor	F-value	p-value	Significance
BU RSD – RollerPressure	6.90	0.034	Significant
BU RSD – RollerSpeed	0.01	0.893	Not Significant
BU RSD – RollerGap	0.20	0.657	Not Significant
CU AV – RollerPressure	6.22	0.041	Significant
CU AV – RollerSpeed	0.65	0.810	Not Significant
CU AV – RollerGap	1.11	0.304	Not Significant
Bulk Density – RollerPressure	0.72	0.417	Not Significant

Bulk Density – RollerSpeed	1.66	0.239	Not Significant
Bulk Density – RollerGap	0.16	0.698	Not Significant
Tapped Density – RollerPressure	1.46	0.266	Not Significant
Tapped Density – RollerSpeed	3.45	0.106	Not Significant
Tapped Density – RollerGap	0.01	0.932	Not Significant

Table S3. Model Adequacy Tests

Model	Lack of Fit p-value	Residuals Pattern	Remarks
BU RSD	Not assessed	Approximately random	Low R^2, not significant
CU AV	Not assessed	Slight curvature	Moderate R^2, borderline significant
Bulk Density	Not assessed	Random	Low fit
Tapped Density	Not assessed	Random	Moderate R^2
